# Recent trends: Retractions of articles in the oncology field

**DOI:** 10.1016/j.heliyon.2024.e33007

**Published:** 2024-06-14

**Authors:** Quan Qi, Jiaqun Huang, Yinhang Wu, Yuefen Pan, Jing Zhuang, Xi Yang

**Affiliations:** a Huzhou Central Hospital, Affiliated Central Hospital Huzhou University, Zhejiang Province, China; b Huzhou Central Hospital, Fifth School of Clinical Medicine of Zhejiang Chinese Medical University, China; c Key Laboratory of Multiomics Research and Clinical Transformation of Digestive Cancer of Huzhou, Zhejiang Province, China

**Keywords:** Retracted articles, CiteSpace, Bibliometric analysis

## Abstract

**Background:**

In recent years, there has been a surge in media reports on articles being retracted after publication. This issue has gained significant attention, particularly due to the consecutive large-scale retractions carried out by renowned international publishers, which have aroused widespread concern in the society.

**Objective:**

To analyze the data of retracted articles and retraction trends.

**Methods:**

The publications were searched through Web of Science Core Collection (WoSCC) database and imported into CiteSpace in plain text format, and visual analysis of countries, institutions, keywords, and subject areas were performed to reveal the trends of retracted articles and the worst areas of retraction.

**Results:**

From 1990 to 2022, 21,568 retracted articles were retrieved, among which the number of retracted articles increased year by year. China is the country with the highest number of retracted articles; Islamic Azad University is the institution with the highest number of retracted articles. In the analysis of all retracted articles across different subject areas, the number of retracted articles in the field of oncology was the highest; In the keyword cluster analysis of retracted articles within the field of oncology, the most prominent category of retracted articles were related to pancreatic cancer.

**Conclusions:**

Scientific and systematic analysis of retracted articles is conducive to improving the quality of papers, raising the level of human research, and cleaning up the research environment.

## 1 Introduction

Retraction is a situation where academic papers are withdrawn by a journal or voluntarily withdrawn by authors after publication due to academic misconduct, errors, or other reasons. Meanwhile, retraction is a worldwide phenomenon as authors from multiple countries of origin involve in research misconduct [1]. The phenomenon of retraction, basically an academic “picketing” of the increasingly visible academic misconduct that began in the 1980s, is one of the most common forms of fabrication, falsification, and plagiarism (“FFP phenomenon”) in the field of academic research. The rules and regulations of academic research are self-correcting regimens established by the academic circle to maintain the integrity of the academic research field. Such rules and regulations are becoming increasingly common and standardized in the field of academic research. The number and frequency of scientific paper retractions are important indicators of the health of the scientific and technical publishing industry, and thus the status of retractions deserves detailed study. The issue of retraction has become a research hotspot for scholars at home and abroad in recent years, and current retractions show at least three characteristics: (1) There is now sufficient evidence that the proportion of published research withdrawn from the scientific literature is rapidly increasing [2]; (2) Withdrawals have occurred in many countries, not only in traditional scientific and technological powerhouses such as the U.S., Germany, and Japan, but also in countries such as China, India, and South Korea, and withdrawals have been extended to “all academic disciplines”; (3) Various reasons for retractions have been identified, which can be broadly categorized into two types, namely misconduct (e.g., falsification of experimental data, duplication of publication, and plagiarism) and unintentional or honest mistakes.

In recent years, the issue of scientific research integrity has attracted worldwide attention. From the viewpoint of disciplines, the problem of retraction in the biomedical field is particularly prominent, and the large-scale retraction of Tumor Biology has shocked the academic community at home and abroad. Therefore, there is an urgent need to analyze the status of paper retraction in recent years and observe and analyze the dynamic evolution of the types of academic misconduct. In this study, retracted articles were searched from January 1, 1990 to December 31, 2022, so as to conduct a detailed study on the retraction situation in recent years, explore the main reasons for retraction, and put forward some suggestions for improvement. The purpose is to provide references for the policy formulation of scientific research academic integrity and basis for the purification of academic environment and the construction of scientific research integrity.

## 2 Methods

### 2.1 Data source and retrieval strategy

The Web of Science (WOS) core database from Clarivate Analytics is deemed as the best for bibliometric analysis, so it was used as the data source. In addition, the WOS core database was searched on September 20, 2023, for all retracted articles. All related publications are collected primarily based on title (T1) using the following search formula: TI=(retract*). Retracted articles from 1990 (January 1, 1990) to 2022 (December 31, 2022) were searched. Retracted articles in the discipline of oncology were collected primarily using the following search formula: TS= (neoplasm* OR tumor* OR neoplasia* OR cancer* OR malignant neoplasm* OR malignancy OR malignancies OR benign neoplasm*). Articles in oncology that were retracted from 2008 (January 1, 2008) to 2022 (December 31, 2022) were searched.

**Fig. 1:**
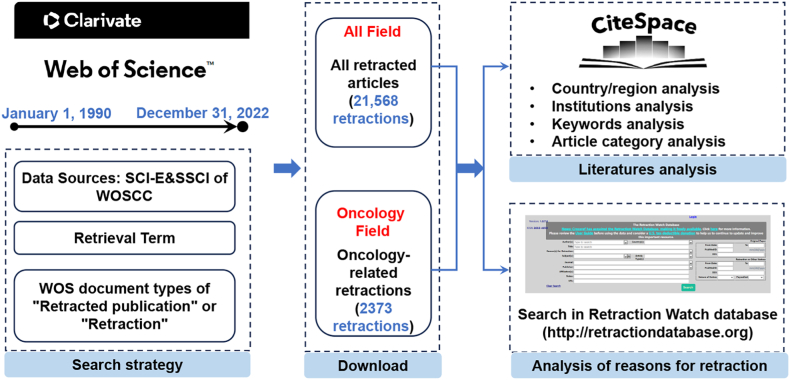
Flowchart of literature screening.

The selection criteria and literature selection process for this study are displayed in Fig. 1. Briefly, the search formula was entered for the initial search and then the publications identified in the initial search reviewed with the following inclusion criteria: (1) publications that have been retracted by the author, institution, editor, or publisher with the help of a WOS document type of “Retracted publication” (meaning a paper that has been “retracted” by the author, institution, editor, or publisher) and “Retracted Paper” (meaning a paper that was created in 2021 and was only assigned when a retraction notice was issued) to analyze the status of retracted articles in WOS; (2) the publication was obtained from the WoSCC Citation Index Expanded (SCI-E) and Social Science Citation Index (SSCI) databases; (3) to avoid bias caused by daily database updates, relevant literature was searched and screened on the same day.

### 2.2 Citespace analysis

Citespace (6.2.R1) software, a literary visualization application created by Professor Chen Chaomei, is a citation visualization analysis software gradually developed in the context of scientometrics and data visualization, with the main function of converting large amounts of documentary data into visual maps. The parameters of CiteSpace are set as follows: g-index, g2 ≤ k Σi ≤ gci, k ∈ Z, k = 25. In the generated graph, N represents the number of network nodes, E is the number of connection lines, Density marks the network density, and Modularity refers to the evaluation index of network modularity. The larger the Modularity Q value is, the better the clustering effect of the network is. The Modularity Q value > 0.3 indicates that the described cluster structure is important. The Silhouette value is used to measure the homogeneity of the network: the closer it is to 1, the higher the homogeneity of the network is, and greater than 0.5 indicates that the cluster structure is reasonable.

## 3 Results

### 3.1 Analysis of retractions over the years

**Fig. 2:**
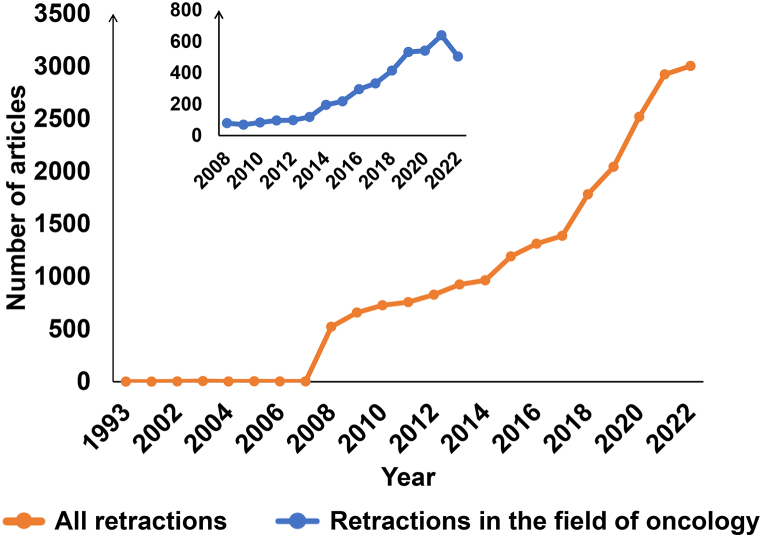
Number of retracted articles over the years. The main graph shows the change of the number of retracted articles over the years for all fields, and the thumbnail shows the change of the number of retracted articles over the years for the oncology subject area.

According to the statistics of articles published from 1990 to 2022, the number of articles has increased year by year since 1990, and the number of retracted articles published in 2022 is the largest, with a total of 3003 retractions (Fig. 2). Since 2008, the number of articles in the field of oncology has increased year by year. 2021 is the year with the highest number of retractions in oncology, with a total of 639 retractions (Fig. 2).

### 3.2 Country/region visual analysis

**Fig. 3:**
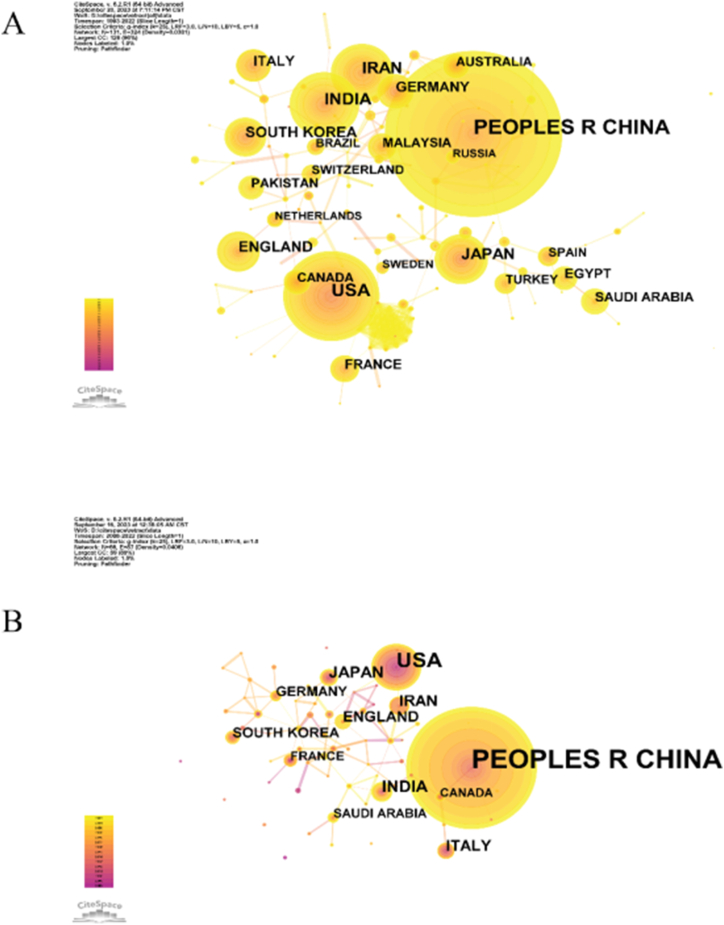
Diagram of the collaborative network of country/regional (A) Analysis of all retracted articles; (B) Analysis of retracted articles in oncology. Nodes represent country lines connecting them. The number of publications is proportional to the size of the nodes. Connections between nodes stand for partnerships. The color changes from purple to yellow from 1990 to 2022.

As of December 31, 2022, among the 21,568 SCI retractions, China had 6,608, far higher than the second place in the United States (2079 articles), India (1166 articles), Iran (907 articles), Japan (703 articles), England (515 articles), South Korea (479 articles), and Germany (388 articles). Chinese retractions accounted for 30.6 % of the global retractions. After a trough of 143 retractions in 2012, the number of retractions in China continued to grow again, reaching 1019 articles in 2020 (Fig. 3A).

**Fig. 4:**
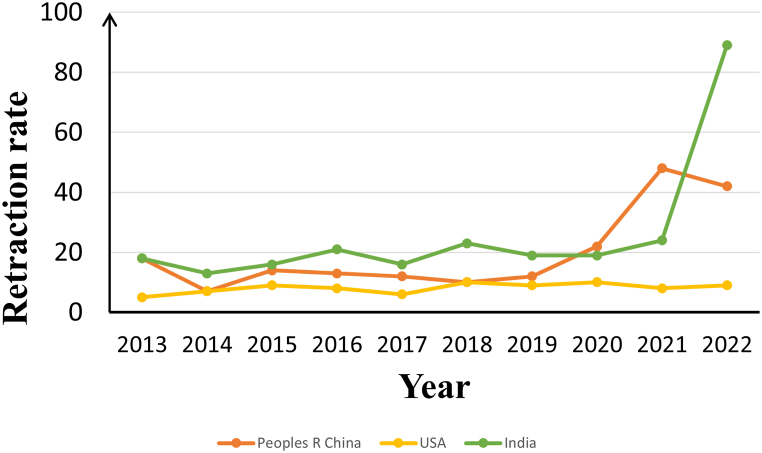
The retraction rate of articles per 10,000 published articles in China, the United States, and India (2013–2022).

The three countries with the highest number of retractions in the last decade are China, the United States, and India. Combining the number of articles published in the three countries in the last decade leads to a question worth exploring: whether the high number of retractions is due to the high volume of publications. As shown in Fig. 4, the United States has a relatively low overall retraction rate of 5–10 retractions per 10,000 publications; India shows the highest retraction rate of 13–89 retractions per 10,000 publications; and China's retraction rate is in the middle of the range, with 7–48 retractions per 10,000 publications. It is important to emphasize that, unlike India's rising retraction rate, China's retraction rate rises significantly in 2020 and 2021 when the global research community was facing unprecedented challenges, and when the pressure and competition for scientific research have increased significantly. Due to limited time and resources in their pursuit of publication numbers, some researchers may have failed to adequately ensure the credibility of their research. Moreover, the Chinese research community's self-correction of articles has led to an increase in the retraction rate in that year. However, it is worth noting that China's retraction rate in 2022 had a downward trend. This may reflect the Chinese research community's attention to and active response to the retraction issue, which is expected to alleviate the retraction problem to a certain extent.

In the field of oncology, the country with the most retractions is still China (2266 articles), followed by the United States (399 articles), India (95 articles), Japan (88 articles), Iran (75 articles), Italy (69 articles) (Fig. 3B).

### 3.3 Visual analytics for research institutions

**Fig. 5:**
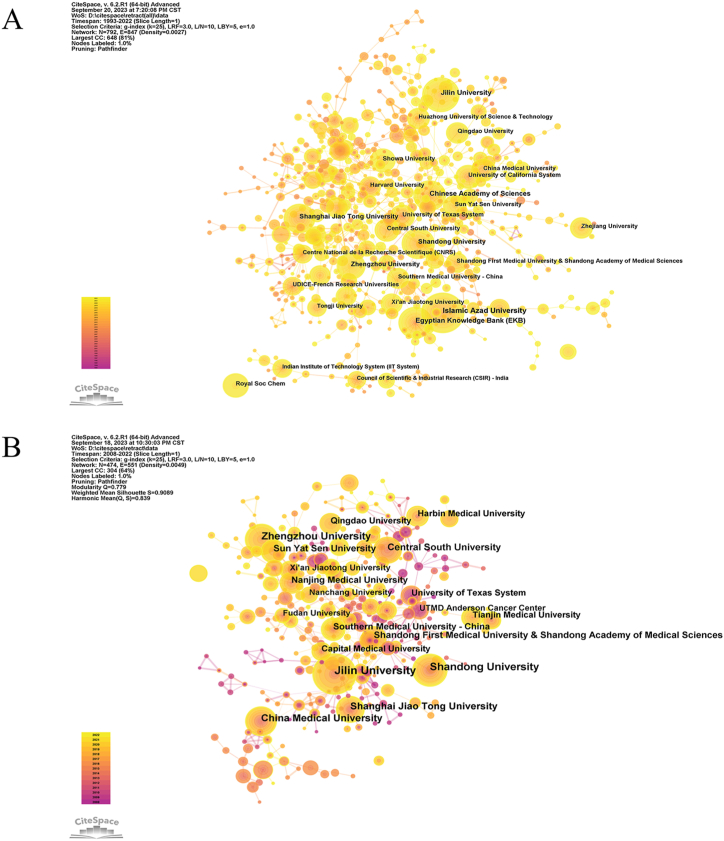
Diagram of the collaborative network of institutional (A) Analysis of all retracted articles; (B) Analysis of retracted articles in oncology. Nodes represent institutions and lines connect them. The number of publications is proportional to the size of the nodes. Connections between nodes mark partnerships.

In the analysis of retraction institutions, Islamic Azad University ranked first (388 retractions), followed by Jilin University (305 articles), Egyptian Knowledge Bank (EKB) (250 articles), Chinese Academy of Sciences (245 articles), and Shandong University (241 articles) (Fig. 5A).

An analysis of institutions with retracted articles on oncology proves that the institution with the most retractions is Jilin University (182 articles), followed by Shandong University (120 articles) and Zhengzhou University (107 articles) (Fig. 5B).

### 3.4 Subject areas and keywords visualization

**Fig. 6:**
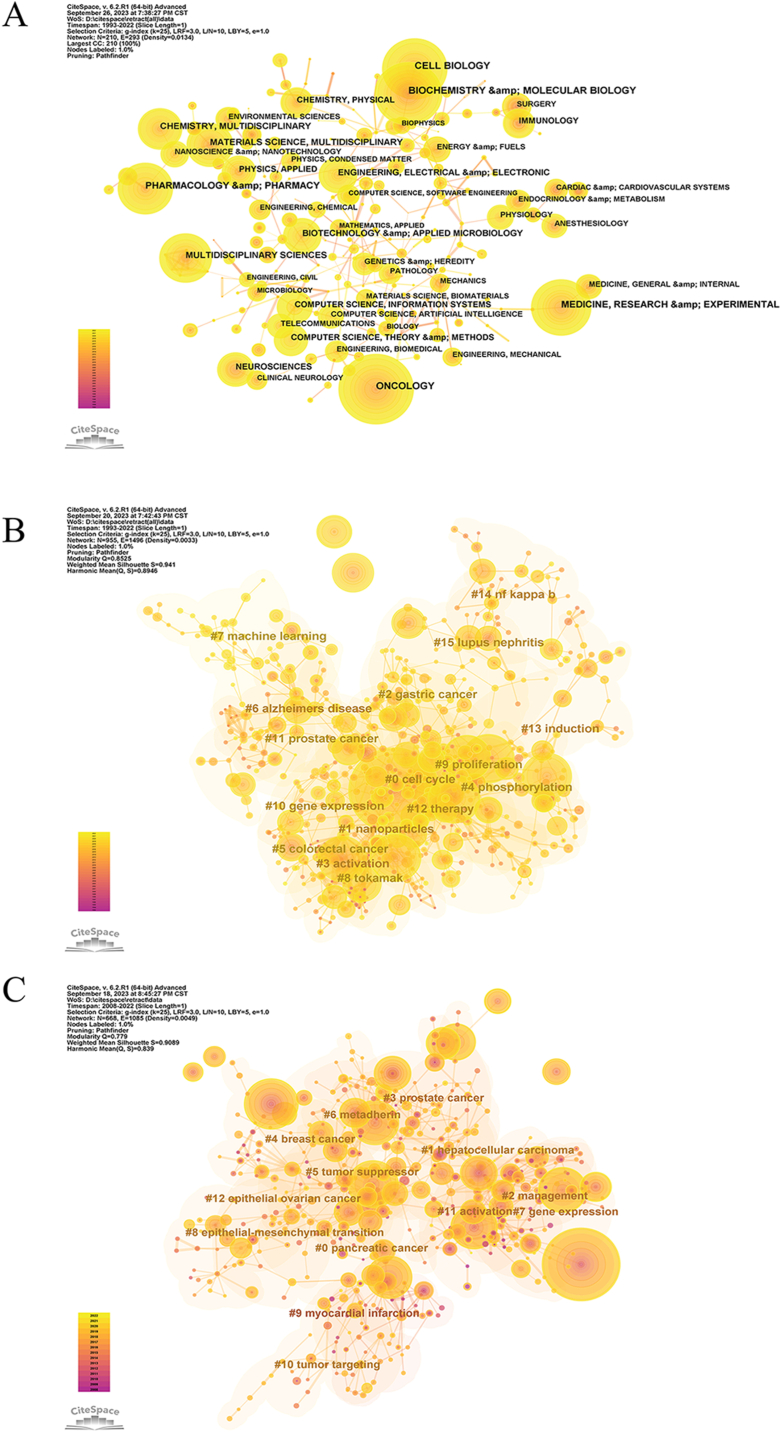
Subject areas and keywords visualization (A) Analysis of subject areas of all retracted articles; (B) Keyword clustering view of all retracted articles; (C) Keyword clustering view of tumor subject areas. Different colors represent different clusters. Each node represents a keyword and the number on the node is the cluster to which the keyword belongs. Labels are assigned to clusters. The smaller the count is, the more keywords are in the cluster.

An analysis of all retracted articles by subject area (an article may contain multiple subject categories for the possible cross-cutting of subject themes in the article) shows that the highest number of articles were retracted in the field of oncology (2373 articles), followed by biochemistry & molecular biology (1931 articles) (Fig. 6A).

The keyword clustering map focuses on reflecting the structural features between clusters, highlighting their key nodes and important connections: “gastric cancer” (#2), “activation” (#3), “phosphorylation” (#4). “colorectal cancer” (#5), “alzheimers disease” (#6), “machine learning " (#7), “tokamak” (#8), “proliferation” (#9), “gene expression " (#10), “prostate cancer” (#11), “therapy” (#12), “induction " (#13), “nf kappa b" (#14), “lupus nephritis” (#15) (Fig. 6B).

Additionally, keyword analysis of retracted articles in the field of oncology was performed, and the keywords were divided into 13 clusters, namely “pancreatic cancer” (#0), “hepatocellular carcinoma " (#1), “management” (#2), “prostate cancer” (#3), “breast cancer " (#4), “tumor suppressor” (#5), “metadherin” (#6), “gene expression " (#7), “epithelial-mesenchymal transition” (#8), “myocardial infarction” (#9), “tumor targeting” (#10), “activation” (#11), and “epithelial ovarian cancer " (#12). The largest cluster was labeled pancreatic cancer (#0), which indicated that the field of pancreatic cancer has the most retracted articles (Fig. 6C).

### 3.5 Reasons for retraction of retracted articles

The WOS database does not have an overall analysis of the reasons for retraction of retracted articles in its own database. Therefore, one needs to read the retraction statement of each paper to understand the description of the reasons for retraction to know the reasons for retraction. Therefore, we searched in Retraction Watch database (http://retractiondatabase.org), which sorted out and classified the reasons for the retractions), for the reasons for each retracted article. All the retractions mainly included the following 29 reasons. Author Unresponsive, Breach of Policy by author or third party, Complaints about author/company/institution/third party, Concerns/Issues About authorship/data/image/referencing/attributions/results, Conflict of interest, Contamination of cell lines/tissues/materials (general)/reagents, Duplication, Error by third party or journal/publisher, Error in analyses/cell lines/tissues/data/image/materials (general)/methods/results/conclusions, Ethical Violations by author/third Party, Euphemisms for duplication, Misconduct, Plagiarism, Manipulation of image or results, Fake Peer Review, Falsification/Fabrication, Investigation by company/institution/journal/publisher/ORI/third party, Hoax paper, Lack of Approval, Legal Reasons/Legal Threats, Miscommunication, Objections, No Further Action, Results Not Reproducible, Retract and Replace, Temporary Removal, Unreliable data/image/results, Paper Mill, Updated to Correction/Retraction.

Among all retractions, concerns/issues with peer review and investigation by journal/publisher/third Party are more common. For example, one published in Applied bionics and biomechanics in 2022 titled “Medical Data Analysis of Lumbar Disc Herniation Patients after. Traditional Chinese Medicine Rehabilitation Intervention Lumbar Function Recovery ", and one published in PloS one in 2013 titled “Mapping C-terminal transactivation domains of the nuclear HER family receptor tyrosine kinase. HER3".

In addition, a range of issues involving author misconduct and scientific dishonesty, mainly including plagiarism, falsification/fabrication, fake peer review, paper mills, were considered the most common reasons for retractions. An article published in The Indian journal of medical research in 2021 titled “Honeycomb-like appearance on optical coherence tomography of the right coronary artery” was retracted with the reason of plagiarism of article. The above behaviours can be mainly manifested as errors and fabrications in methods and results, which make the results difficult to reproduce and articles are judged to be unreliable. An article published in Experimental eye research titled “LncRNA MIR7-3HG executes a positive role in retinoblastoma progression via modulating miR-27a-3p/PEG10 axis” was retracted due to error in methods and results not reproducible. An article published in British journal of pharmacology titled “Role of hypoxia-inducible factors in the dexrazoxane-mediated protection of cardiomyocytes from doxorubicin-induced toxicity” was retracted due to duplication of image and original data not provided. An article published in Biomedicine & pharmacotherapy titled “MicroRNA-30b promotes lipopolysaccharide-induced inflammatory injury and alleviates autophagy through JNK and NF-κB pathways in HK-2 cells” was retracted due to concerns/issues about data, false/forged authorship, investigation by third party, paper mill, and results not reproducible. An article published in Molecular biology reports titled “Genetic variations in the KIR gene family may contribute to susceptibility to ankylosing spondylitis: a meta-analysis” was retracted due to fake peer review.

The reasons for the high number of retractions in oncology are as follows. First, due to the large number of topics and scientific research projects in the field of oncology, the number of published literatures is large. According to Pubmed database, in 2023, there were more than 230,000 articles in the field of oncology worldwide. In addition, in order to ensure the completion of the project, researchers have falsified experimental data and other problems of scientific dishonesty, which increased the risk of article retraction [3,4]. Taken in the field of oncology draft articles, duplication, paper mill, fake peer review, falsification/fabrication and misconduct has been found to be the most common cause. And two or more of these problems can exist simultaneously. For instance, “PEA15 promotes liver metastasis of colorectal cancer by upregulating the ERK/MAPK signaling pathway” published in Oncology reports was retracted as a result of duplication of image and paper mill. “miR-484 suppresses proliferation and epithelial-mesenchymal transition by targeting ZEB1 and SMAD2 in cervical cancer cells” published in Cancer cell international was retracted as a result of concerns/issues about data and duplication of image. “MicroRNA-1271 functions as a potential tumor suppressor in hepatitis B virus-associated hepatocellular carcinoma through the AMPK signaling pathway by binding to CCNA1” published in Journal of cellular physiology was retracted due to duplication of image and paper mill. “ALDH2 and ADH1 Genetic Polymorphisms May Contribute to the Risk of Gastric Cancer: A Meta-Analysis” published in PloS one was retracted due to error in methods, fake peer review and paper mill. “Low-dose human atrial natriuretic peptide for the prevention of postoperative cardiopulmonary complications in chronic obstructive pulmonary disease patients undergoing lung cancer surgery” published in European journal of cardio-thoracic surgery was retracted due to falsification/fabrication of Data and misconduct by author.

## 4 Discussion

The guidelines for retraction on the official website of the International Committee on Publication Ethics (COPE) suggest two main reasons for retraction [5], namely genuine human error, such as errors in experimental data or statistical analysis, and academic misconduct, such as fabrication, hidden conflicts of interest, ethical violations, and plagiarism. The latter is known to be more serious than the former, but unfortunately, similar cases continue to increase. Fang et al. evaluated the reasons for retractions of articles in the fields of biomedicine and life sciences and found that 67.4 % of the retractions were due to some form of misconduct and only 21.3 % were due to errors [6]. Bozzo et al. [7] focused only on retractions in oncology and determined that 61 % of retractions were due to research misconduct. For example, recently, two major academic journals, Nature [8] and Science [9], withdrew one of their respective articles due to alleged academic fraud by Abderrahmane Kaidi, an author involved in the preparation of both papers, who reported falsified research data.

In the past, published articles mainly relied on journal editors and reviewers to check the authenticity of the results of the research and had peers test the results by repeating the experiments after a paper was published. Since 2010, with the establishment of websites, such as Retraction Watch and PubPeer, scholarly publications have been subject to more direct scrutiny, questioning, and tracking [10]. As a result, an increasing number of articles have been retracted for falsification/fabrication of data, duplication of articles, or lack of IRB/IACUC approval [11]. Some studies found that the top 5 reasons for retraction include Duplication of Image, Paper Mill, Fake Peer Review, Duplication of Article, Falsification/Fabrication, and Falsification/Fabrication [11,12]. In 2023, more than 10,000 research papers were withdrawn, creating the largest number of retractions in history. Among them, Hindawi, a subsidiary of Wiley, accounted for the majority of retractions, with more than 8000 articles. Over the past 20 years, Saudi Arabia had the most retractions, followed by Pakistan, Russia, and China [3]. Burhan et al. found that plagiarism, error and duplication were common reasons for retractions of biomedical literature, and oncology ranked among the top 3 retractions of biomedical literature [13]. Recently, apart from the above common reasons for retraction, artificial intelligence technologies, such as ChatGPT, are also important [14]. The retracted papers did not label AI-assisted writing in their articles. In addition, ChatGPT and AI-assisted writing software tools generate fake references, while these AI-assisted articles passed peer review, which resulted in a deeper problem. Peer review experts often do not have enough time to thoroughly review manuscripts to discern whether there are signs of AI use. The rapid rise of artificial intelligence has provided ample ammunition for “paper factories” to produce low-quality and unscientific articles [15,16]. Some articles were submitted and published without the knowledge or consent of the principal investigator (PI), and the author voluntarily requested the retraction of the article [17]. One study found that the number of oncology retractions published by Chinese scholars from 2013 to 2022 was as high as 2695. The retractions were published from 2017 to 2020, and research articles accounted for 94.17 %. The top three reasons for retractions were data, results and image problems, duplicate publication and paper factory [18].

Overall, academic misconduct remains the main reason for retractions. There are many underlying reasons behind scientific misconduct, including the lack of ethical self-discipline of some researchers, insufficient research training for a significant number of researchers and clinicians, inadequate research management in the organization, and problems in assessment and evaluation. In this regard, the relevant departments have taken some targeted institutional measures [19,20]. First, all parties should strengthen the education and training of researchers in integrity. Second, a favourable research environment and academic ecology should be further created. Third, universities and scientific research organizations should strengthen the inspection and supervision of research results. Fourth, investigations of scientific research misconduct should be coordinated and transparent. These measures and policies will undoubtedly help the scientific community to better self-correct, ensure the credibility and quality of research results, and further increase public trust and support the scientific enterprise.

In the present study, 32 years of retractions in WOS database were included, and Citespace was used for literature analysis to reveal the current situation and development trend of retractions in a more scientific way. This study could guide significance in maintaining academic integrity and establish a scientific, reasonable and targeted scientific research standard system. In terms of limitations, since this study involved more than 20,000 retractions, WOS database did not mark the reasons for the retractions, so there was no detailed analysis.

## Funding statement

This research received no specific grant from any funding agency in the public, commercial or not-for-profit sectors.

## Data availability statement

N/A.

## CRediT authorship contribution statement

**Quan Qi:** Conceptualization. **Jiaqun Huang:** Data curation. **Yinhang Wu:** Visualization. **Yuefen Pan:** Conceptualization. **Jing Zhuang:** Writing – original draft. **Xi Yang:** Writing – review & editing.

## Declaration of competing interest

The authors declare that they have no known competing financial interests or personal relationships that could have appeared to influence the work reported in this paper.

## References

[1] Amos K.A. (2014). The ethics of scholarly publishing: exploring differences in plagiarism and duplicate publication across nations. J. Med. Libr. Assoc..

[2] Cokol M., Ozbay F., Rodriguez-Esteban R. (2008). Retraction rates are on the rise. EMBO Rep..

[3] Van Noorden R. (2023). More than 10,000 research papers were retracted in 2023 - a new record. Nature.

[4] Pantziarka P., Meheus L. (2019). Journal retractions in oncology: a bibliometric study. Future Oncol..

[5] Wager E., Barbour V., Yentis S., Kleinert S. (2009). Retractions: guidance from the Committee on publication ethics (COPE). Croat. Med. J..

[6] Fang F.C., Steen R.G., Casadevall A. (2012). Misconduct accounts for the majority of retracted scientific publications. Proc. Natl. Acad. Sci. U.S.A..

[7] Bozzo A., Bali K., Evaniew N., Ghert M. (2017). Retractions in cancer research: a systematic survey. Res Integr Peer Rev.

[8] Kaidi A., Jackson S.P. (2019). Retraction Note: KAT5 tyrosine phosphorylation couples chromatin sensing to ATM signalling. Nature.

[9] Kaidi A., Weinert B.T., Choudhary C., Jackson S.P. (2010). RETRACTED: human SIRT6 promotes DNA end resection through CtIP deacetylation. Science.

[10] Didier E., Guaspare-Cartron C. (2018). The new watchdogs' vision of science: a roundtable with Ivan oransky (retraction Watch) and brandon stell (PubPeer). Soc. Stud. Sci..

[11] Wang B., Lai J., Yan X., Jin F., Yao C. (2020). Exploring the characteristics, global distribution and reasons for retraction of published articles involving human research participants: a literature survey. Eur. J. Intern. Med..

[12] Lei L., Zhang Y. (2018). Lack of improvement in scientific integrity: an analysis of WoS retractions by Chinese researchers (1997-2016). Sci. Eng. Ethics.

[13] Kocyigit B.F., Akyol A. (2022). Analysis of retracted publications in the biomedical literature from Turkey. J. Kor. Med. Sci..

[14] Rahimi F. (2023). Talebi bezmin abadi A: **passive contribution of ChatGPT to scientific papers**. Ann. Biomed. Eng..

[15] Conroy G. (2023). Scientific sleuths spot dishonest ChatGPT use in papers. Nature.

[16] Conroy G. (2023). How ChatGPT and other AI tools could disrupt scientific publishing. Nature.

[17] Wu H., Luan Y. (2023). Retraction: achieving near-Pt hydrogen production on defect nanocarbon via the synergy between carbon defects and heteroatoms. Chem. Commun..

[18] Yang W., Sun N., Song H. (2024). Analysis of the retraction papers in oncology field from Chinese scholars from 2013 to 2022. J. Cancer Res. Therapeut..

[19] Horbach S., Bouter L.M., Gaskell G., Hiney M., Kavouras P., Mejlgaard N., Allum N., Aubert Bonn N., Bendtsen A.K., Charitidis C.A. (2022). Designing and implementing a research integrity promotion plan: recommendations for research funders. PLoS Biol..

[20] Laar A.K., Redman B.K., Ferguson K., Caplan A. (2020). Institutional approaches to research integrity in Ghana. Sci. Eng. Ethics.

